# Investigation of the Effects of the Short QT Syndrome D172N Kir2.1 Mutation on Ventricular Action Potential Profile Using Dynamic Clamp

**DOI:** 10.3389/fphar.2021.794620

**Published:** 2022-01-18

**Authors:** Chunyun Du, Randall L. Rasmusson, Glenna C. Bett, Brandon Franks, Henggui Zhang, Jules C. Hancox

**Affiliations:** ^1^ School of Physiology, Pharmacology and Neuroscience, University of Bristol, Bristol, United Kingdom; ^2^ Department of Physiology and Biophysics, Jacobs School of Medicine and Biomedical Sciences, University of New York, University at Buffalo, Buffalo, NY, United States; ^3^ Cytocybernetics Inc, North Tonawanda, NY, United States; ^4^ Department of Obstetrics and Gynecology, Center for Cellular and Systems Electrophysiology, State University of New York, University at Buffalo, Buffalo, NY, United States; ^5^ Biological Physics Group, Department of Physics and Astronomy, The University of Manchester, Manchester, United Kingdom

**Keywords:** arrhythmia, dynamic clamp, KCNJ2, Kir2.1, short QT syndrome, ventricular myocyte

## Abstract

The congenital short QT syndrome (SQTS) is a cardiac condition that leads to abbreviated ventricular repolarization and an increased susceptibility to arrhythmia and sudden death. The SQT3 form of the syndrome is due to mutations to the *KCNJ2* gene that encodes Kir2.1, a critical component of channels underlying cardiac inwardly rectifying K^+^ current, I_K1_. The first reported SQT3 *KCNJ2* mutation gives rise to the D172N Kir2.1 mutation, the consequences of which have been studied on recombinant channels *in vitro* and in ventricular cell and tissue simulations. The aim of this study was to establish the effects of the D172N mutation on ventricular repolarization through real-time replacement of I_K1_ using the dynamic clamp technique. Whole-cell patch-clamp recordings were made from adult guinea-pig left ventricular myocytes at physiological temperature. Action potentials (APs) were elicited at 1 Hz. Intrinsic I_K1_ was inhibited with a low concentration (50 µM) of Ba^2+^ ions, which led to AP prolongation and triangulation, accompanied by a ∼6 mV depolarization of resting membrane potential. Application of synthetic I_K1_ through dynamic clamp restored AP duration, shape and resting potential. Replacement of wild-type (WT) I_K1_ with heterozygotic (WT-D172N) or homozygotic (D172N) mutant formulations under dynamic clamp significantly abbreviated AP duration (APD_90_) and accelerated maximal AP repolarization velocity, with no significant hyperpolarization of resting potential. Across stimulation frequencies from 0.5 to 3 Hz, the relationship between APD_90_ and cycle length was downward shifted, reflecting AP abbreviation at all stimulation frequencies tested. In further AP measurements at 1 Hz from hiPSC cardiomyocytes, the D172N mutation produced similar effects on APD and repolarization velocity; however, resting potential was moderately hyperpolarized by application of mutant I_K1_ to these cells. Overall, the results of this study support the major changes in ventricular cell AP repolarization with the D172N predicted from prior AP modelling and highlight the potential utility of using adult ventricular cardiomyocytes for dynamic clamp exploration of functional consequences of Kir2.1 mutations.

## Introduction

Cardiac action potential (AP) repolarization depends on the dynamic interplay between multiple ionic currents, including those carried by a number of key potassium channels (Varro et al., 2020). Thus, early repolarization of ventricular APs is influenced by the rapidly activating and inactivating transient outward potassium current, I_TO_ (Tamargo et al., 2004; Varro et al., 2020), whilst the rapid and slow delayed rectifier potassium currents (I_Kr_ and I_Ks_) contribute to repolarization over AP plateau voltages (Mitcheson and Sanguinetti, 1999; Tamargo et al., 2004; Varro et al., 2020). The terminal phase of ventricular AP repolarization is substantially driven by the inwardly rectifying K^+^ current, I_K1_ (Tamargo et al., 2004; Varro et al., 2020). Early application of the AP voltage clamp technique to rabbit ventricular myocytes showed that I_K1_ is suppressed at AP plateau voltages, but increases steeply during the late phase of the AP, peaking negative to ∼ −60 mV, before then declining as the AP voltage approaches the K^+^ equilibrium potential (Shimoni et al., 1992). From work on guinea-pig ventricular myocytes Ishihara and Ehara suggested that the increase in outward I_K1_ during repolarization involves relief of block of the underlying channels by intracellular Mg^2+^ ions (Ishihara and Ehara, 1998). The relative timing of ventricular I_Kr_ and I_K1_ ensures that as the former current declines, the latter increases, assuming the dominant role in final repolarization (Mitcheson and Hancox, 1999; Banyasz et al., 2011; Horvath et al., 2021). Functional I_K1_ channels are comprised of tetramers of Kir2.x α subunits (Dhamoon and Jalife, 2005; Hibino et al., 2010; Varro et al., 2020). *KCNJ2* is responsible for Kir2.1 which is strongly expressed in human atria and ventricles (Wang et al., 1998; Gaborit et al., 2007). The importance of Kir2.1 in contributing to native I_K1_ is demonstrated by the fact that loss-of-function *KCNJ2* mutations have been implicated in Andersen-Tawil syndrome, which causes one form of long QT syndrome (long QT type 7) as well as extra-cardiac abnormalities (Tristani-Firouzi and Etheridge, 2010; Perez-Riera et al., 2021). Conversely, over-expression of Kir2.1 in the mouse heart produces a substrate favourable to high-frequency rotor development and rotor stabilization (Noujaim et al., 2007). Moreover, gain-of-function *KCNJ2* mutations have been implicated in human familial atrial fibrillation (Xia et al., 2005) and the SQT3 variant of the short QT syndrome (SQTS; Priori et al., 2005; Hattori et al., 2012; Deo et al., 2013; Ambrosini et al., 2014).

The congenital SQTS is characterized by abbreviated QT intervals, (often) tall upright T waves and increased susceptibility to ventricular arrhythmias (Maury et al., 2008; Hancox et al., 2018). The SQT3 *KCNJ2* missense mutations thus far identified impair Kir2.1 current rectification, with the consequence that outward I_K1_ is augmented and thereby makes a greater contribution to AP repolarization (Hancox et al., 2018; Hancox et al., 2019). The first reported SQT3 Kir2.1 mutation (D172N) was identified in an asymptomatic 5-year old girl with an abnormal electrocardiogram, whose father had a history of presyncopal events and palpitations (Priori et al., 2005). Both father and daughter had markedly abbreviated QT_c_ intervals (320 ms) and asymmetric T waves (Priori et al., 2005). The D172 residue is located in the Kir2.1 pore and is involved in polyamine and Mg^2+^ block of the channel that gives rise to voltage dependent rectification (Abrams et al., 1996; Priori et al., 2005). The D172N mutation impairs this process, resulting in a preferential augmentation of outward current (Priori et al., 2005; El Harchi et al., 2009). Simulations incorporating the consequent change to I_K1_ have shown action potential and QT interval shortening and increased tissue vulnerability to atrial and ventricular arrhythmias (Priori et al., 2005; Adeniran et al., 2012; Whittaker et al., 2017). The M301K and E299V Kir2.1 SQT3 mutations impair Kir2.1 current rectification to greater extents than does D172N (Hattori et al., 2012; Deo et al., 2013).

There are at present no genotypically accurate experimental models of SQT3 and therefore information on the functional consequence of SQT3 mutations has almost exclusively derived from electrophysiological studies of recombinant channels and computational modelling (Priori et al., 2005; El Harchi et al., 2009; Adeniran et al., 2012; Hattori et al., 2012; Deo et al., 2013; Ambrosini et al., 2014; Whittaker et al., 2017). Patient-specific human induced pluripotent stem cell derived cardiomyocytes (hiPSC-CMs) have recently been used to study *KCNH2* mutation linked SQT1 variants of the SQTS (El-Battrawy et al., 2018; Guo et al., 2018). However, iPSC-CMs tend to exhibit an immature spontaneously active phenotype in which I_K1_ is sparse or absent (Doss et al., 2012; Hoekstra et al., 2012) and so are not as readily used to study mutations that affect I_K1_. Adenoviral transduction with Kir2.1 provides one potential solution to this problem (Lieu et al., 2013; Jones et al., 2014), although precise titration of wild-type and mutant channel subunits to compare homozygous and heterozygous expression conditions represents a technical challenge. An alternative approach is to supply a “virtual” I_K1_ electronically using the “dynamic clamp” method (Bett et al., 2013). The real-time application of virtual I_K1_ has been shown to improve hiPSC-CM AP configuration in both manual and automated patch clamp approaches (Bett et al., 2013; Meijer van Putten et al., 2015; Goversen et al., 2017; Verkerk et al., 2017; Becker et al., 2020). Moreover, Meijer van Putten and others adopted this approach to show hiPSC-CM AP changes with simulated loss of I_K1_ (mimicking Andersen-Tawil syndrome) and of increased I_K1_ due to the effects of the E299V SQT3 mutation (which produced marked AP abbreviation; Meijer van Putten et al., 2015). Although hiPSC-CMs have the advantage of their human origin, they nonetheless retain the drawback of lack of maturity. Consequently, a complementary approach is the use of adult ventricular myocytes from an appropriate model species. Accordingly, the present study was undertaken to investigate the effects on adult guinea-pig ventricular myocyte AP configuration of real-time replacement of a wild-type I_K1_ with SQT3 (D172N) I_K1_, using WT and mutant formulations for I_K1_ previously derived from experimental data and used for *in silico* cell and tissue models (El Harchi et al., 2009; Adeniran et al., 2012; Whittaker et al., 2017). Our findings constitute a direct demonstration of causality between the D172N Kir2.1 mutation and abbreviated ventricular AP repolarization.

## Methods

### Guinea-Pig Ventricular Myocyte Isolation

Adult male guinea-pigs (300–600 g) were killed in accordance with UK Home Office legislation. Left ventricular myocytes were then isolated by enzymatic and mechanical dispersion as described previously (Zhang et al., 2001; Ridley et al., 2003). Briefly, guinea-pigs were anaesthetized with pentobarbital sodium (140 mg/kg, I.P.) together with heparin (4,000 U/kg). The heart was removed quickly and was then cannulated and perfused with a Langendorff perfusion system at 37°C. The basic perfusion solution contained: 130 NaCl, 5.4 KCl, 5 HEPES, 10 Glucose, 0.4 NaH_2_PO_4_, 3 MgCl_2_, 20 taurine and 10 creatine (pH 7.61 with NaOH). First, basic solution containing 750 μM of CaCl_2_ was perfused for 2 min, then Ca^2+^-free basic perfusion solution was supplied for 5 min, followed by low Ca^2+^ (150 μM) containing basic solution that also contained collagenase (Worthington, Type I, 0.3 mg/ml per100 g body weight) and protease (Merck, Type XIV 0.01 mg/ml per100 g body weight) for 4–7 min. Cells were then released from left ventricle by mechanical dispersion. Isolated myocytes were stored in low calcium solution (150 μM) at room temperature. Aliquots of the cell suspension were transferred into a chamber mounted on an inverted microscope and left to settle for several minutes, before being exposed to normal Tyrode solution. Only cells with a clear rod-shape and striated appearance were chosen for recording. The mean cell capacitance of guinea-pig ventricular myocytes used in this study was 98.3 ± 12.5 pF (12 myocytes from 8 hearts).

### Culture of Cor.4U Human Induced Pluripotent Stem Cell Derived Cardiomyocytes

Cor.4U hiPSC-CMs were obtained from Axiogenesis (Nattermannallee, Köln, Germany). The cells were cultured according to the manufacturer’s instructions for manual patch clamp experiments (MPC Protocol V1.1). Briefly, cells were thawed and suspended in culture medium (Axiogenesis) and seeded (20 μl at concentration of 5*10^4^ cells/ml) on gelatin (0.1%) coated glass shards in 12-well plates. After incubation at 37°C in 5% CO_2_ for 3 h, the culture medium was added to the plates. The cells were kept in a 5% CO_2_ incubator at 37°C with culture medium changed twice a week. Whole-cell patch clamp was conducted in weeks 2–3 of cell culture. The mean cell capacitance of hiPSC-CMs in this study was 56.8 ± 4.8 pF (n = 14 cells).

### Electrophysiological Recording

Aliquots of adult guinea-pig left ventricular myocytes or glass shards with hiPSC-CMs were placed on the glass bottom of the chamber mounted on the microscope and the cells were continuously superfused at 37 ± 1°C with a standard Tyrode’s solution containing (in mM): 140 NaCl, 4 KCl, 2 CaCl_2_, 1 MgCl_2_, 10 Glucose, 5 HEPES (titrated to pH 7.4 with NaOH; osmolarity 292 mOsm/L). For guinea-pig myocyte experiments, glass-pipettes (Corning 7,052 glass, AM Systems Inc.) were pulled with a horizontal micropipe puller (Sutter, P-97I) to give a resistance of 1–2 MΩ. For stem cell experiments, glass-pipettes were pulled with a vertical puller (Narishige, PP 830) and polished (Narishige, MF83) to give a final resistance of 2–3 MΩ. The pipette dialysis solution contained (in mM): 110 KCl, 10 NaCl, 5 MgATP, 0.4 MgCl_2_, 10 HEPES, 5 Glucose. Pipette pH (and thereby bulk intracellular pH) was buffered to pH 7.2 with 10 mM HEPES (titrated with KOH, increasing pipette [K^+^] to 120 mM; osmolarity 248 mOsm/L). The theoretical potassium equilibrium potential (E_K_) for these external and pipette solutions was −90.85 mV at 37°C. Series resistance values (typically 2–5 MΩ) were compensated by >70%. Transmembrane currents and action potentials were recorded in the whole-cell mode using an Axopatch 1D amplifier (Molecular Devices) and a CV-4 1/100 head stage. Data were recorded via a Digidata 1200B interface and stored on the hard-disk of a Viglen computer. Data digitization rates were 10 kHz during all protocols and an appropriate bandwidth of 2 kHz was set on the amplifier. Data acquisition (Clampex 8.2) and analysis (Clampfit 10.7) were performed using pCLAMP (Molecular Devices), Excel 365, Origin (2018 b) and GraphPad Prism (8) Software.

### Dynamic Clamp

Dynamic clamp was carried out using a commercially available Cybercyte dynamic clamp system (Cybercyte V10) (Bett et al., 2013). The simulation system of the Cybercyte dynamic clamp consisted of a 16 channel, 16 bit, 100 kS/s MCC PCIe-DAS1602/16 board installed and configured in a Dell 5,810 Precision workstation. The average measured loop time for the system was 46 µs. The extracellular K concentration was set to 4 mM.

The model formulations for I_K1_ were based on the formulations of Adeniran et al. (2012) and are as follows:

$$I_{K1}=G_{K1}\sqrt{\frac{K_{o}}{5.4}}X_{K1\infty }\left(V-E_{K}\right)$$

Where $X_{K1\infty }=\frac{\alpha_{K1}}{\alpha_{K1}+\beta_{K1}}$

WT

$$\alpha_{K1}=\frac{0.07}{1+e^{0.017\left(\text{V}-E_{K}-200.2\right)}}$$

$$\beta_{K1}=\frac{3e^{0.0003\left(\text{V}-E_{K}+100.2\right)}+e^{0.08\left(\text{V}-E_{K}-8.7\right)}}{1+e^{-0.024\left(\text{V}-E_{K}\right)}}$$

$$G_{K1}=4.8\;nS/pF$$

WT-D172N

$$\alpha_{K1}=\frac{0.1}{1+e^{0.023\left(\text{V}-E_{K}-199.9\right)}}$$

$$\beta_{K1}=\frac{3e^{0.0002\left(\text{V}-E_{K}+100.4\right)}+e^{0.07\left(\text{V}-E_{K}-9.8\right)}}{1+e^{-0.021\left(\text{V}-E_{K}\right)}}$$

$$G_{K1}=8.26\;nS/pF$$

(1.72 fold the WT value).

D172N

$$\alpha_{K1}=\frac{0.1}{1+e^{0.05\left(\text{V}-E_{K}-199.9\right)}}$$

$$\beta_{K1}=\frac{3e^{0.0002\left(\text{V}-E_{K}+100.1\right)}+e^{0.08\left(\text{V}-E_{K}-10.3\right)}}{1+e^{-0.006\left(\text{V}-E_{K}\right)}}$$

$$G_{K1}=10.75\;nS/pF$$

(2.24 fold the WT value).

G_K1_ is the maximal conductance of I_K1_; the values given above are exemplar starting values. In practice, the WT amplitude was scaled to produce an APD_90_ in Ba^2+^-conditions that matched that in control Tyrode’s solutions and the values for WT-D172N and D172N were then scaled accordingly (see next section). X_K1∞_ is the time-independent inward rectification factor that is a function of voltage; $\sqrt{\frac{K_{o}}{5.4}}$ is the K_o_ dependence of the current.

### Testing the Dynamic Clamp Output

**FIGURE 1:**
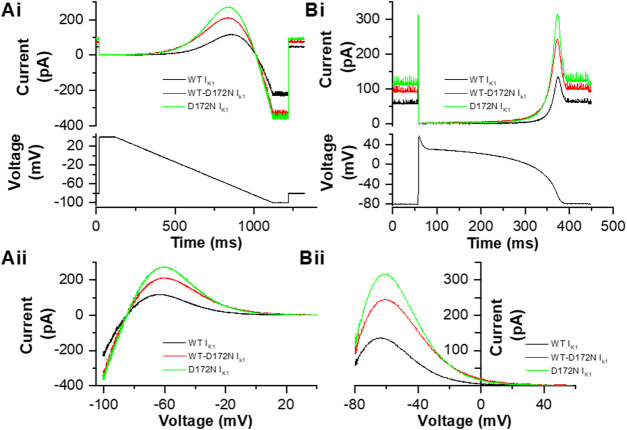
I_K1_ formulations employed in this study. **Ai** Example current traces of WT I_K1_ (black), WT-D172N I_K1_ (red) and D172N I_K1_ (green) current derived from a conventional descending voltage ramp protocol shown in the lower panel, recorded from a model cell. **Aii**. I–V relations for WT, WT-D172N and D172N conditions. Peak outward I_K1_ during the descending voltage ramp occurred at −63.5, −60.5, and −60 mV respectively. **Bi.** Example current traces of WT I_K1_ (black), WT-D172N I_K1_ (red) and D172N I_K1_ (green) current derived from an action potential (AP) protocol shown in the lower panel, recorded from a model cell. **Bii.** Instantaneous I–V relations for WT, WT-D172N and D172N conditions during the AP repolarization phase (repolarization occurs from right to left on this plot). Peak outward current during AP repolarization occurred at −64, −60.5, and −60 mV respectively.

**FIGURE 2:**
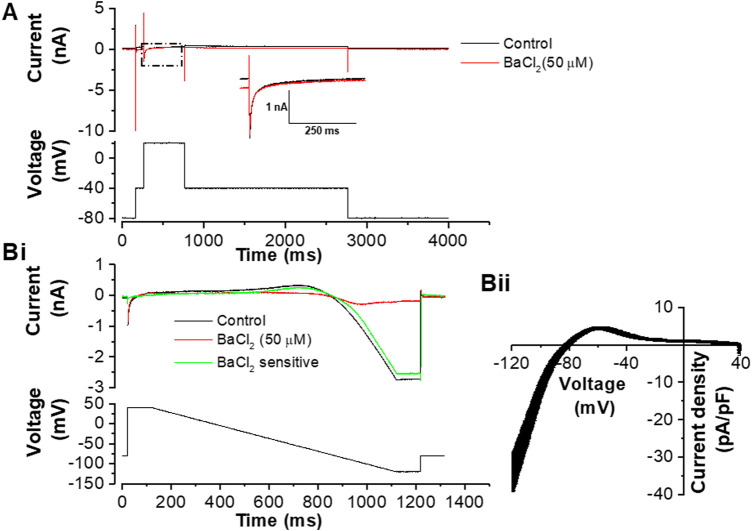
Effects of 50 µM BaCl_2_ on voltage clamped guinea-pig ventricular myocytes. **(A)**. Exemplar current traces (upper) elicited by the protocol shown in the lower panel. From a holding potential of −80 mV a step to −40 mV was applied to activate and inactivate fast sodium current (I_Na_); this was followed by a step to +20 mV which elicited L-type Ca current (I_Ca,L_). The black trace is in control and the red trace is in the presence of 50 µM BaCl_2_. The inset shows expanded records of the portion of the traces marked by the dashed box, in order to aid visualization of I_Ca,L._ **Bi**. Example current traces elicited by the protocol shown in the lower panel. The black trace is in control, red trace is with 50 µM of BaCl_2_ and green trace is the BaCl_2_ sensitive current. **Bii**. Mean ± SEM I-V plot of Ba^2+^-sensitive current from 11 cells from 8 hearts (mean cell capacitance of 97.6 ± 13.7 pF). The measured reversal potential of this current was −81.6 ± 0.8 mV.

Prior to application of the dynamic clamp I_K1_ formulations to ventricular myocytes, an experiment was conducted in which the I_K1_ formulations to be used were applied in voltage clamp mode to a model cell (comprised of a 10 MΩ resistor and 500 MΩ resistor in series with one another, in parallel with a 33 pF capacitor in cell mode). Figure 1Ai shows WT, D172N and WT-D172N currents elicited by a descending voltage ramp (+40 to −100 mV over 1s). The Cytocybernetics system allows 1) current amplitude to be scaled as necessary during an experiment in order to provide an output of appropriate size; 2) the ratio of the different formulations to be fixed, so that these match experimentally derived values. Here, peak outward I_K1_ for D172N-WT was set to be 1.72 the WT current, whilst that for D172N was set to be 2.24 that for the WT current (estimated from Figure 1A of (Adeniran et al., 2012)). The corresponding current-voltage (I-V) relations are shown in Figure 1Aii, with I_K1_ reversing at −85 mV. This reversal potential for the I_K1_ formulation used in dynamic clamp was considered appropriate as it is close to that of−83.1 ± 0.6 mV reported for guinea-pig ventricular I_K1_ from perforated patch recordings by Ishihara et al. (2002) and is also near the reversal potential for Ba^2+^ sensitive I_K1_ in Figure 2 of this study. As seen experimentally (Priori et al., 2005; El Harchi et al., 2009), outward current was larger and peaked at a more positive voltage for the D172N than the WT condition (by +3.5 mV compared to WT), with the D172N-WT condition intermediate in amplitude between the two (peak shifted by +3 mV compared to WT). Once these ratios had been set, an action potential (AP) clamp waveform was applied to the Cybercyte I_K1_ models of each condition using a simulated human ventricular AP waveform from the ten Tusscher et al. model (Ten Tusscher et al., 2004; McPate et al., 2009). The current outputs from the models during the AP command are shown in Figure 1Bi. As the holding potential (−80 mV) lay positive to E_rev_ there was outward holding current prior to the AP upstroke phase. A brief transient current was apparent during the AP upstroke and then little or no current flowed through part of the AP plateau, with a marked increase during late repolarization. Figure 1Bii shows the instantaneous I-V relation for I_K1_ during AP repolarization, with marked increases in current late in repolarization and current peaking at a more positive voltage for D172N than WT current, with D172N-WT being intermediate (similar to the ramp current in Figure 1Aii and *cf* El Harchi et al., 2009). These observations confirmed that the current formulations could be applied in real-time and were as expected for the WT and mutant conditions to be studied subsequently under dynamic clamp.

Data are presented as mean ± SEM. Statistical tests used to evaluate statistical significance are given in the relevant Results text and were conducted using Microsoft Excel (Office 365), GraphPad PRISM 8.4.3 or GraphPad Instat 3.10. *p* < 0.05 was considered statistically significant.

## Results

### Effects of Barium Block of I_K1_ on Ventricular AP Profile

In order to be able to replace endogenous I_K1_ with virtual I_K1_ via dynamic clamp, it was necessary to utilize an inhibitor of I_K1_. In early experiments we attempted to use the pentamidine analogue PA-6 (1 μM; (Takanari et al., 2013)), but in our hands I_K1_ inhibition was slow to develop (*cf* Sanson et al., 2019) making it unsuitable for the purposes of this study. Instead, a low concentration of barium (Ba^2+^) ions was used to inhibit I_K1_. Native I_K1_ has been reported to be inhibited by Ba^2+^ with an IC_50_ of 4.7 µM (Schram et al., 2003) and the concentration used here (50 µM) was sufficient to block I_K1_ substantially without altering the amplitude or time-course of L-type calcium current (I_Ca,L_) (Figure 2). Figure 2A shows representative I_Ca,L_ records on step depolarization from −40 to +20 mV, with a K^+^-based pipette dialysate: current during the step to +20 mV was unaltered, whereas the time-independent current at −40 mV was diminished, as expected for outward I_K1_ blockade. Figure 2Bi shows current during a descending ramp protocol in the absence and presence of Ba^2+^ ions, whilst Figure 2Bii shows the current-voltage (I-V) relation for Ba^2+^-sensitive I_K1_ elicited by the ramp protocol. The Ba^2+^ sensitive I_K1_ density observed with this protocol at −120 mV was similar to that reported previously for guinea-pig ventricular I_K1_ elicited by a voltage step protocol (James et al., 2004).

**FIGURE 3:**
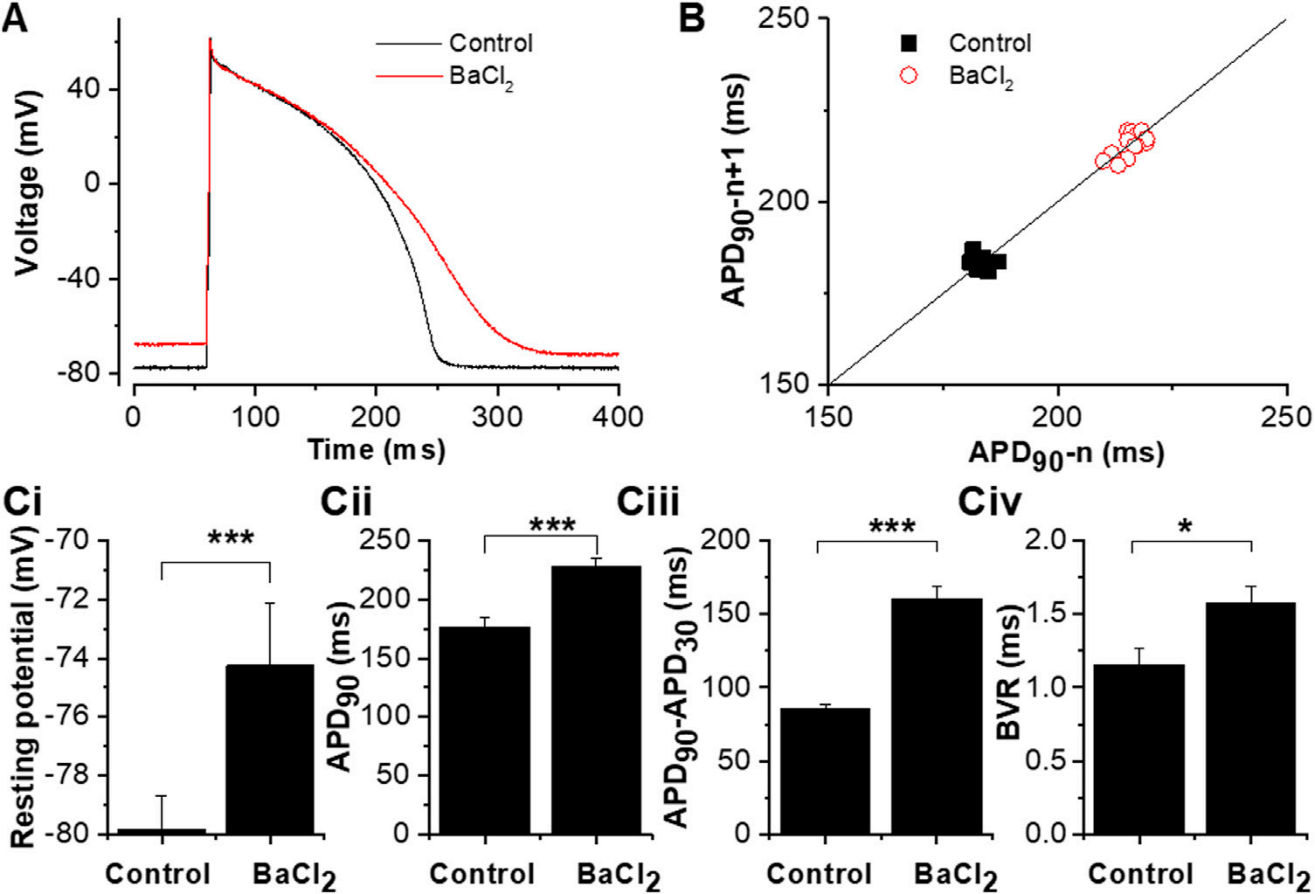
Effects of 50 µM BaCl_2_ on the guinea-pig ventricular action potential. **(A)**. The effect of 50 µM BaCl_2_ on action potentials elicited from guinea-pig ventricular myocyte by brief (3 ms) suprathreshold (1700 pA) current pulses applied at 1 Hz (black trace in control and red trace in 50 µM BaCl_2_). **(B)**. Poincaré plots of APD_90_ from 15 continuously action potentials showing the effect of BaCl_2_ on beat-to-beat variability. Solid line shows line of identity (x = *y*). **Ci.** Effects of 50 µM BaCl_2_ on the measured resting potential. **Cii.** Effects of 50 µM BaCl_2_ on APD_90_ (for each cell, average APD_90_ values from 5 APs in each of control and Ba^2+^ conditions were obtained and then pooled). **Ciii**. Effects of 50 µM BaCl_2_ on action potential triangulation (measured as the difference between APD_90_ and APD_30_). **Civ.** Effects of 50 µM BaCl_2_ on beat-to-beat variability [beat-to-beat variability was quantified as APD_90_ variability: $\sum \left(\left|APD_{90\left(n+1\right)}-APD_{90n}\right|\right)/\left(n*\sqrt{2}\right)$ , where APD_90n_ and APD_90(n+1)_ indicated the APD_90_ durations of the *n*th and (n+1)th APs respectively. n denoted the number of consecutive bests analysed (15 here)]. “*” denotes statistical significance of *p* < 0.05; “***” denotes statistical significance of *p* < 0.001 (paired *t*-test, n = 12 cells from 8 hearts).

Figure 3 shows the effect of Ba^2+^ ion application on the guinea-pig ventricular AP configuration and resting potential. For these experiments brief (2–10) ms suprathreshold depolarizing currents were applied to elicit APs (determined for each cell by progressive increase in current amplitude and or duration, up to a maximum of 10 ms in control conditions, then set to 100–200 pA above the threshold). Figure 3A shows representative APs elicited at 1 Hz (using a 3 ms injection of 1700 pA depolarizing current) in the absence and presence of Ba^2+^. Control and +Ba^2+^ APs were closely superimposed early during repolarization, but they diverged later in the AP plateau phase and terminal repolarization. The Poincaré plot in Figure 3B shows measurements of beat-to-beat variability (BVR) in APD_90_ in control solution and in the presence of I_K1_ inhibition by Ba^2+^: variability appeared to be greater in the latter condition. Figure 3C shows mean data from 12 similar experiments. Resting potential (Figure 3Ci) was significantly depolarized in the presence of Ba^2+^ by ∼ 6 mV (5.6 ± 1.1 mV; n = 12). APD_90_ was significantly increased (Figure 3Cii) and the difference between APD_90_ and APD_30_ (a measure of AP triangulation (Hondeghem et al., 2001)) was also significantly augmented by Ba^2+^ application. Evaluation of the APD_30_/APD_90_ ratio from the same experiments was also consistent with augmented AP triangulation by Ba^2+^ ions (Control ratio of 0.51 ± 0.02 and of 0.31 ± 0.03 with Ba^2+^ application; *p* < 0.0001, paired *t*-test). BVR in APD was quantified as shown in Figure 3Civ (Valentin et al., 2004; Hancock et al., 2015) and was found to be significantly greater in the presence of Ba^2+^. To summarise: I_K1_ inhibition with Ba^2+^ ions depolarized resting potential, increased APD_90_ and AP triangulation and augmented BVR.

### Effects of the D172N Mutation Studied With Dynamic Clamp of Guinea-Pig Ventricular Myocytes

For experiments investigating the effects of the D172N mutation, for baseline APs the Ba^2+^-inhibited I_K1_ was replaced by WT virtual I_K1_, scaling the magnitude of I_K1_ to restore APD_90_. In 12 cells from 8 animals, APD_90_ in control Tyrode was 177.3 ± 8.7 ms; once endogenous I_K1_ had been inhibited by Ba^2+^ and replaced in dynamic clamp, APD_90_ was 183.9 ± 9.3 ms (*p* > 0.05 (*p* = 0.09) vs control Tyrode). The resting potential was also restored (−79.8 ± 1.1 mV in control Tyrode and −78.5 ± 0.8 mV in Ba^2+^+WT virtual I_K1_; *p* > 0.05 (*p* = 0.17)). For these experiments, we also measured the peak outward I_K1_ inhibited by Ba^2+^ ions during a voltage ramp protocol (460.8 ± 42.6 pA; 4.9 ± 0.4 pA/pF) and compared this with the peak outward virtual I_K1_ supplied in dynamic clamp to restore APD_90_ (599.2 ± 76.4 pA; 6.5 ± 0.8 pA/pF). Although there was a trend for the latter to be greater, this was not statistically significant (*p* > 0.05 for both absolute currents and current densities; paired *t*-test).

**FIGURE 4:**
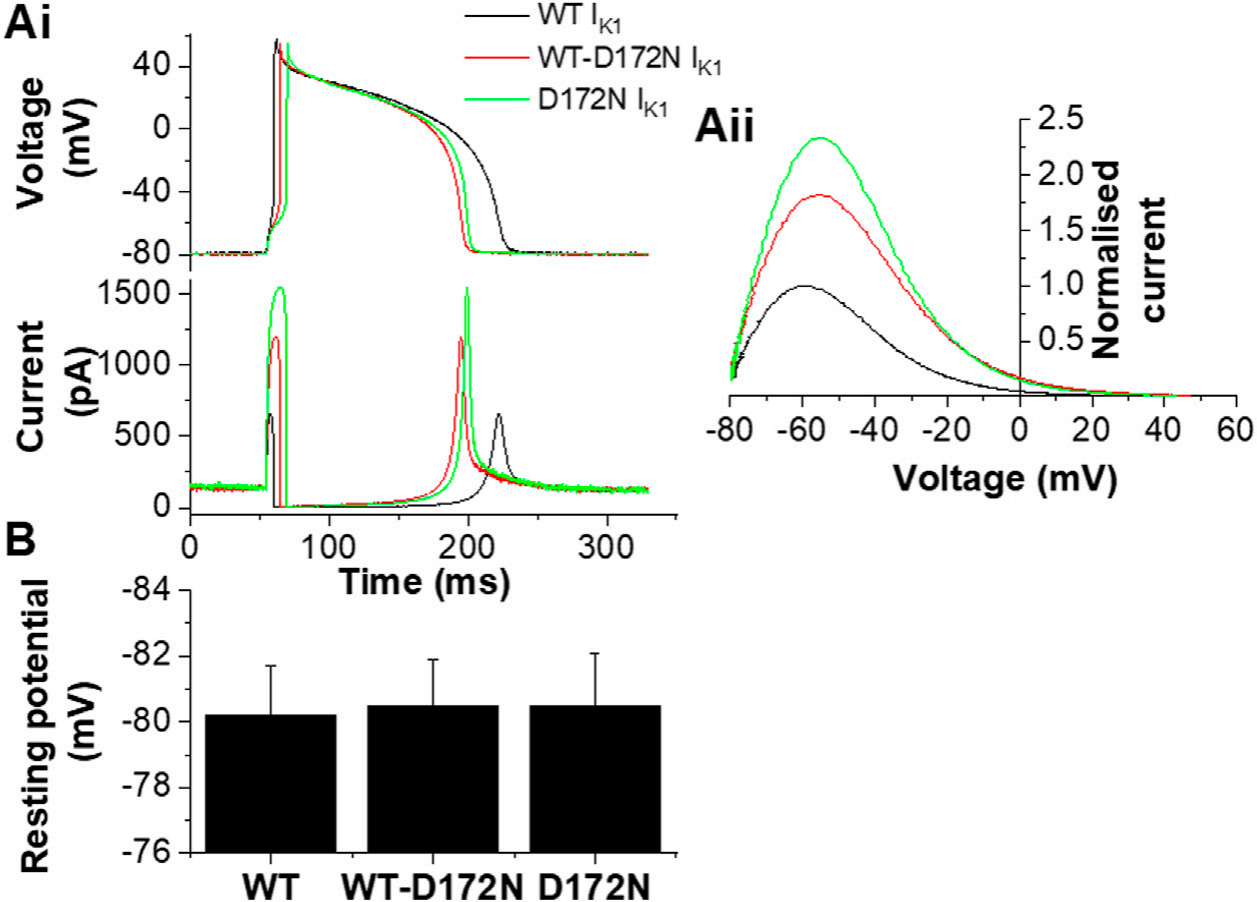
Guinea-pig ventricular APs under dynamic clamp. **Ai**. Guinea-pig ventricular APs elicited from a cell by brief suprathreshold current pulses (applied at 1Hz; 1800 pA for 5, 10 and 14 ms for WT, WT-D172N and D172N conditions respectively). Upper traces show APs and lower traces show corresponding applied I_K1_. Black traces correspond to WT I_K1_, red traces correspond to WT-D172N I_K1_, green traces correspond to D172N I_K1_. **Aii.** Instantaneous I–V relations for WT, WT-D172N and D172N conditions during the AP repolarization phase (from 10 ms after the AP peak to completion of repolarization; repolarization occurs from right to left on this plot. Currents were normalized to peak outward current with the WT I_K1_ formulation). Peak outward current during AP repolarization occurred at −61.5, −57 and −57 mV respectively. **(B)**. Effects of different I_K1_ formulations on AP resting potential of guinea-pig ventricular myocytes (n = 5 cells from 4 hearts to which each of WT, WT-D172N and D172N I_K1_ were applied).

**FIGURE 5:**
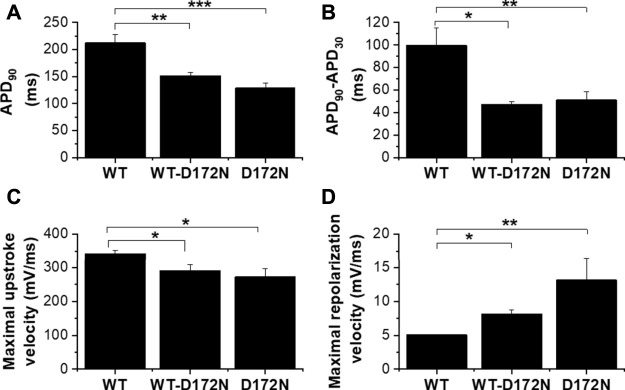
Mean effects of different I_K1_ formulation on guinea-pig ventricular action potential parameters. **(A)**. Mean (±SEM) of APD_90_ values for APs elicited at 1 Hz for WT, WT-D172N and D172N I_K1_ conditions. **(B)**. Mean (±SEM) APD_90_-APD_30_ values (as an index of AP triangulation) for WT, WT-D172N and D172N I_K1_ conditions. **(C)**. Mean (±SEM) maximal upstroke velocity (V_max_) values for WT, WT-D172N and D172N I_K1_ conditions. **(D)**. Mean (±SEM) maximal repolarization velocity values for WT, WT-D172N and D172N I_K1_ conditions. “*” denotes statistical significance of *p* < 0.05; “**” denotes statistical significance of *p* < 0.01; “***” denotes statistical significance of *p* < 0.001 (one-way ANOVA test, followed by Tukey’s multiple comparisons test; n = 5 cells from 4 hearts to which all I_K1_ formulations were applied).

The upper panel of Figure 4Ai shows exemplar AP traces at 1 Hz with WT, WT-D172N and D172N I_K1_. The brief depolarizing current pulses supplied to elicit APs were altered as required when the WT I_K1_ formulation was replaced with WT-D712N and D172N formulations. Both heterozygotic and homozygotic D172N I_K1_ formulations led to APD abbreviation, associated with increased I_K1_ amplitude during late repolarization. The mean charge (product of current amplitude and duration) supplied to elicit APs was 8.7 ± 1.2 pC, 13.3 ± 2.1 pC and 19.0 ± 3.7 pC for WT, WT-D172N and D172N formulations respectively (n = 5; *p* < 0.05 between both mutation conditions and WT; one way ANOVA with Tukey post-test). Figure 4Aii shows instantaneous I-V relations for I_K1_ during the AP repolarization phase, which closely resembled those seen with the model cell in Figure 1Bii. In addition, a rapid outward I_K1_ transient was seen during the AP that was coincident with the AP upstroke and that differed between WT and mutant conditions (Figure 4Ai). This rapid current component is in line with previous experimental observations: a similar transient outward component of native guinea-pig and canine ventricular I_K1_ has been reported previously in experiments employing more classical voltage clamp protocols (Li et al., 1998; Li et al., 2000) and is visible in some of the dynamic clamp I_K1_ records in hiPSC-CM experiments performed by Meijer van Putten et al (2015). As shown in Figure 4Ai there appeared to be little change in the resting potential between WT and mutant I_K1_ conditions. This observation was borne out by analysis of mean data from 5 similar experiments in which all 3 I_K1_ formulations were successfully applied to each cell, as no significant difference in resting potential was observed (Figure 4B). Figure 5 shows mean AP parameter data from 5 experiments. Figure 5A shows mean APD_90_ values; APD_90_ was significantly abbreviated by both I_K1_ mutant formulations. Replacement of WT I_K1_ with WT-D127N I_K1_ led to a 27.7 ± 2.8% shortening of APD_90_; although there was a trend towards a greater effect of the D172N I_K1_ formulation (38.0 ± 6.1% shortening compared to WT), this was not statistically significantly different from WT-D172N (*p* > 0.05). Switching from WT to WT-D172N I_K1_ led to a marked reduction in APD triangulation (APD_90_—APD_30_, Figure 5B), which was not further significantly altered by switching to D172N I_K1_ alone. Figure 5C shows maximal AP upstroke velocity (V_max_) with WT and mutant formulations; there was a modest but significant reduction in V_max_ compared to WT with both heterozygotic and homozygotic D172N formulations. As the current stimuli used to elicit APs were set beyond threshold levels, it was not possible to ascertain whether or not the reduction in V_max_ was accompanied by any significant change in AP threshold voltage. However, peak overshoot potential values were analysed and found to be: 58.2 ± 3.5 mV, 51.7 ± 2.4 mV and 54.0 ± 3.2 mV (n = 5) for WT, WT-D172N and D172N formulations respectively. Although there was a trend towards a reduction in overshoot amplitude with the mutant I_K1_ formulations, this did not reach statistical significance (Friedman test; *p* = 0.09).

Figure 5D shows maximal repolarization velocity under the 3 different conditions. Both heterozygotic and homozygotic D172N formulations led to significant acceleration of terminal repolarization compared to the WT condition; however, whilst there was a trend towards a greater effect of D172N than WT-D172N, this did not attain significance. BVR was 1.6 ± 0.2 ms in control and 1.3 ± 0.1 ms and 1.7 ± 0.3 ms in heterozygous and homozygous conditions respectively (*p* > 0.05, one-way ANOVA test followed by Tukey’s multiple comparisons test; n = 5 cells from 4 hearts).

**FIGURE 6:**
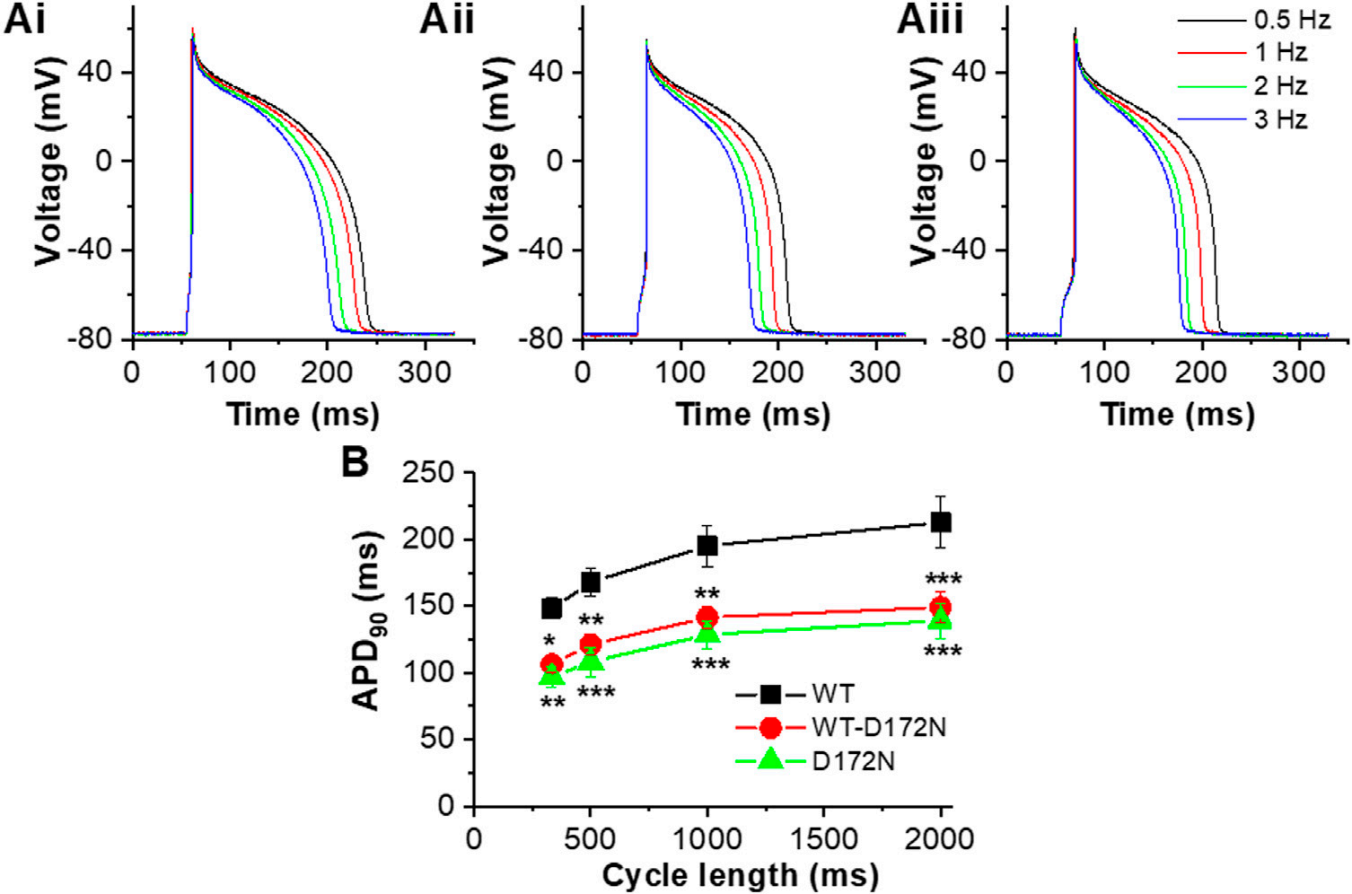
AP rate-dependence for WT, WT-D172N and D172N I_K1_. **(A)**. Example APs elicited in a single experiment at 4 stimulation frequencies (0.5, 1, 2, 3 Hz) with WT I_K1_ **Ai**, WT-D172N I_K1_ **Aii** and D172N I_K1_ **Aiii**. **(B)**. Plot of APD_90_ against cycle length (1/frequency) for WT I_K1_ (black), WT-D172N I_K1_ (red) and D172N I_K1_ (green) (n = 5 cells from 4 hearts). “*” denotes statistical significance against WT of *p* < 0.05; “**” denotes statistical significance against WT of *p* < 0.01; “***” denotes statistical significance against WT of *p* < 0.001 (two-way ANOVA test, followed by Tukey’s multiple comparisons test; n = 5 cells from 4 hearts to which all I_K1_ formulations were applied).

Additional measurements were performed in which APs were elicited at different rates (0.5, 1, 2 and 3 Hz) with the 3 different I_K1_ formulations in turn (exemplar traces are shown in Figure 6A). At least 20 APs at each rate were sampled and APD_90_ values from 5 successive APs at each rate were averaged and pooled to obtain the mean data plots shown in Figure 6B. As anticipated as the cycle length was reduced (with each I_K1_ formulation) APs shortened and APD_90_ was accordingly reduced. Switching from WT I_K1_ to either heterozygotic (WT-D172N) or homozygotic (D172N) mutant conditions led to a marked abbreviation of APD_90_ at all rates tested (*p* < 0.05 for WT-D172N and *p* < 0.001 for D172N vs WT respectively; see Figure 6 legend for significance values from comparison of individual stimulus frequencies between mutant and WT conditions). Thus, introduction of the D172N mutation abbreviated APD_90_ over the entire range of stimulation frequencies examined. There was little difference between the two mutant conditions, however. Due to difficulties in maintaining recordings at high rates, stimulation rates above 3 Hz were not applied and so APD_90_ values at shorter cycle lengths were not obtained.

### Effect of Mutant I_K1_ Formulations on the Configuration of APs From hiPSC-Cardiomyocytes

**FIGURE 7:**
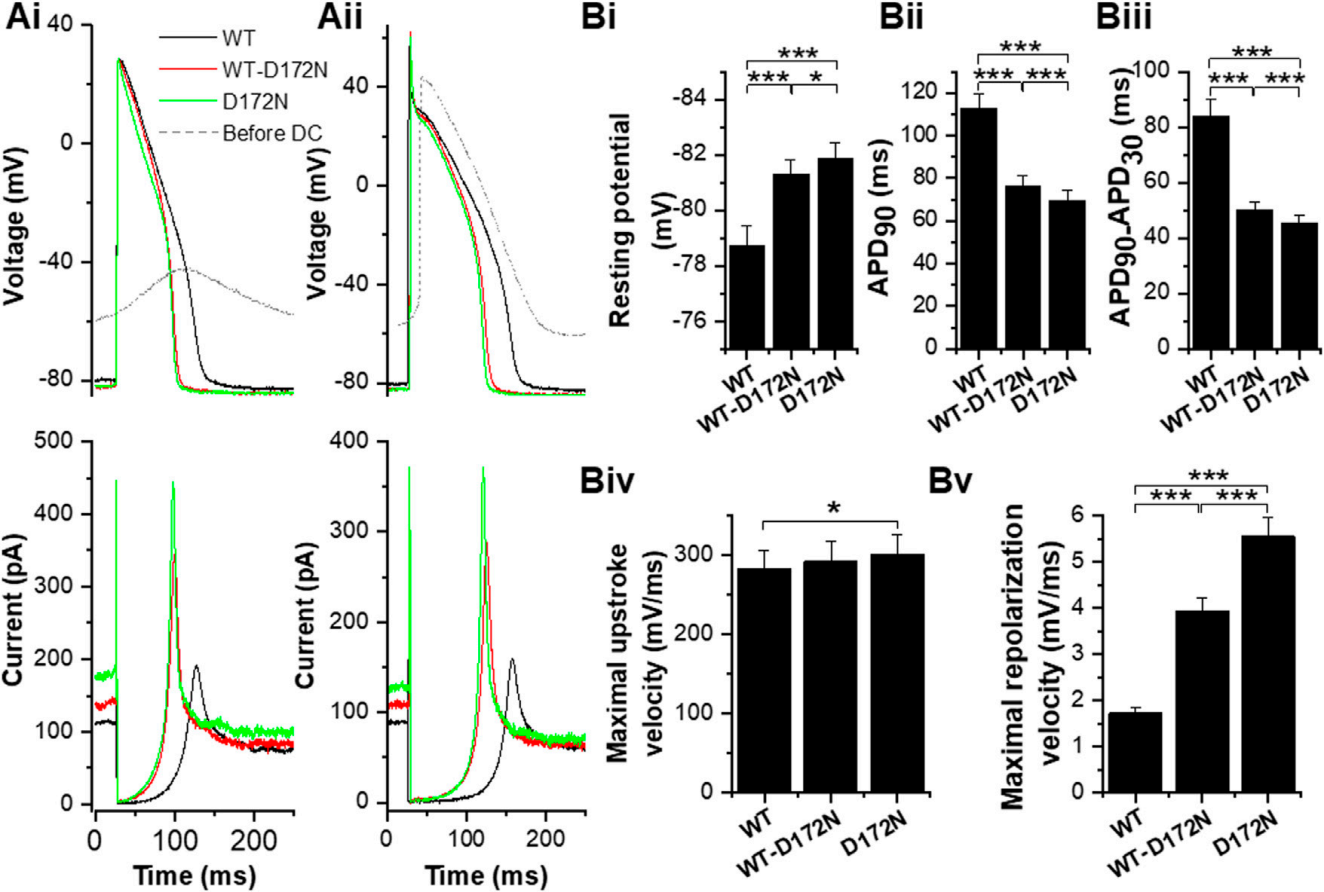
Effects of the D172N-altered I_K1_ on human iPSC-CM APs. **(A)**. Example traces of APs elicited at 1 Hz (by 1.5 ms of 2000 pA current in **Ai**; 1 ms of 2000 pA in **Aii** from iPSC cardiomyocytes to which different I_K1_ formulations were applied (black for WT I_K1_, red for WT-D172N I_K1_ and green for D172N I_K1_; superimposed grey traces shows spontaneously oscillating membrane potential (left cell) and spontaneous AP (right cell) prior to activation of the dynamic clamp). Lower traces show corresponding traces of the I_K1_ supplied in each condition. **Bi.** Mean (±SEM) resting potential values for WT, WT-D172N and D172N I_K1_ conditions. **Bii.** Mean (±SEM) maximal APD_90_ values for WT, WT-D172N and D172N I_K1_ conditions. **Biii.** Mean (±SEM) maximal APD_90_-APD_30_ values (as an index of AP triangulation) for WT, WT-D172N and D172N I_K1_ conditions. **Biv**. Mean (±SEM) maximal upstroke velocity (V_max_) values for WT, WT-D172N and D172N I_K1_ conditions. **Bv.** Mean (±SEM) maximal repolarization velocity values for WT, WT-D172N and D172N I_K1_ conditions. “*” denotes statistical significance of p < 0.05; “**” denotes statistical significance of p < 0.01; “***” denotes statistical significance of p < 0.001 (one-way ANOVA test, followed by Tukey’s multiple comparisons test; n = 14 cells for all).

Although the principal focus of this study was on effects of alterations to I_K1_ on APs from adult guinea-pig ventricular myocytes, for comparative purposes a limited series of experiments were conducted at 1 Hz on human iPSC-CMs. COR-4U cardiomyocytes have only low *KCNJ2* expression (Huo et al., 2017). Unpaced COR-4U myocytes have also been reported to exhibit little or no I_K1_ up to 14 days in culture post thaw, with perinuclear staining of Kir2.1 (Gélinas et al., 2017). Moreover, Ba^2+^-sensitive current recorded from these iPSC-CMs has been reported to have a reversal potential and rectification profile that differ from those expected from a pure I_K1_ (Goversen et al., 2017). The majority of iPSC-CMs from which we recorded (11/14) exhibited spontaneous APs with a mean maximum diastolic potential (MDP) of −54.3 ± 3.6 mV, which would not be expected in the presence of a substantial I_K1_. In consequence, the different I_K1_ formulations were applied in dynamic clamp experiments without prior application of Ba^2+^ ions and with application of sufficient WT I_K1_ to give a ventricular-like AP configuration and stable resting potential close to −80 mV (−78.7 ± 0.7 mV). In 14 cells APs with the WT I_K1_ formulation were elicited by brief depolarizing current injections (supplying 2.5 ± 0.2 pC; *p* < 0.001 vs WT I_K1_ guinea-pig ventricular APs, Mann-Whitney test). The peak outward WT I_K1_ supplied by dynamic clamp during repolarization was 164.4 ± 10.2 pA (3.0 ± 0.1 pA/pF). For each cell, WT-D172N and D172N formulations were then applied at the same fixed ratios as used in guinea-pig myocyte experiments. Changes in the magnitude of the stimulus required to elicit APs were not required when I_K1_ formulations were changed. Figure 7A shows exemplar traces of APs elicited at 1 Hz (upper traces) from two iPSC-CMs, with the corresponding applied WT and mutant I_K1_ also shown (lower traces). For completeness, also superimposed are membrane potential recordings from each cell prior to activation of dynamic clamp. The cell in Figure 7Ai showed a subthreshold oscillating membrane potential, whilst that in Figure 7Aii exhibited spontaneous APs prior to injection of synthetic I_K1_ under dynamic clamp. The I_K1_ profiles under dynamic clamp were qualitatively similar to those seen in guinea-pig myocyte experiments with the clear difference that proportionately greater current was evident during the resting potential [compare, for example, the relative amplitude (ratio) of peak I_K1_ during repolarization with that at the resting potential for the WT I_K1_ records in Figure 4A (a ratio of 4.9) and Figure 7A (ratios of 1.7 and 1.8 in Ai and Aii)]. This presumably reflects the immature phenotype of the hiPSC-CMs and requirement for I_K1_ to establish a stable negative resting potential. In contrast with the observations from guinea-pig myocytes, the resting potential differed between WT, WT-D172N and D172N conditions, becoming progressively more negative as the amplitude of outward I_K1_ increased due to the mutation (Figure 7A, Bi). Although the negative shifts in resting potential were small, they were nevertheless statistically significant. Despite the presence of rapid outward I_K1_ transients during the AP upstroke, upstroke V_max_ did not decrease under mutant conditions (there was no significant difference between WT V_max_ and that with WT-D172N I_K1_, whilst there was a small but significant increase for D172N I_K1_ alone; Figure 7Biv). There was no significant difference in mean overshoot potential between WT, WT-D172N and D172N conditions (53.1 ± 4.2 mV, 51.8 ± 3.7 mV and 51.4 ± 3.5 mV respectively; n = 14, *p* > 0.05 one-way ANOVA). Effects of the D172N mutant I_K1_ formulations on APD_90_ (Figure 7Bii) and AP triangulation (Figure 7Biii) in iPSC-CMs were similar to those seen in guinea-pig myocytes, with the greatest differences seen on switching from WT to WT-D172N I_K1_ formulations, with small (but significant) additional changes on switching to D172N I_K1_ alone. Analysis of maximum repolarization velocity (Figure 7Bv) showed this to be accelerated with the mutant I_K1_ formulations compared to the WT condition.

## Discussion

### Effects of Ba^2+^ and Establishing the WT Baseline

Although multiple studies have now used dynamic clamp to compensate for the deficit of intrinsic I_K1_ in stem-cell derived cardiomyocytes (e.g., Bett et al., 2013; Meijer van Putten et al., 2015; Goversen et al., 2017; Verkerk et al., 2017; Fabbri et al., 2019; Becker et al., 2020) there is relatively little published work in which I_K1_ has been supplied to isolated adult cardiomyocytes from human-relevant model species using this technique. Dynamic clamp has been used in the exploration of regional differences in I_K1_ shape and magnitude in the canine heart (Cordeiro et al., 2015). A recent study has applied synthetic I_K1_ through dynamic clamp to isolated human atrial myocytes (Verkerk et al., 2021): application of I_K1_ with moderate rectification significantly improved resting potentials, whilst the atrial APs with injected I_K1_ exhibited a sensitivity to blockers of other ion channels that indicated that major ionic currents remained functional (Verkerk et al., 2021). The data from our experiments now show that virtual I_K1_ replacement in adult ventricular myocytes using dynamic clamp facilitates study of the consequences of pathological changes in I_K1_ consequent to an underlying gene mutation, as conducted previously for I_Kr_ and an LQT2 mutation (Berecki et al., 2005).

The concentration of Ba^2+^ ions used here to inhibit I_K1_ (50 µM) is the same as used independently in AP clamp studies to isolate guinea-pig and canine I_K1_ (Banyasz et al., 2011; Horvath et al., 2021). We did not expose myocytes in this study to high (mM) Ba^2+^ concentrations in order to minimize the likelihood of affecting other ionic currents involved in AP genesis. Liu et al (2001) reported K_D_ values for Ba^2+^ inhibition of I_K1_ of 0.21–1.14 µM between −120 and −80 mV, whilst Schram and others (2003) reported an IC_50_ for I_K1_ block of 4.7 µM. Using that IC_50_ value, 50 µM Ba^2+^ might be predicted to produce ∼90% inhibition of the current. Ishihara et al (2002) reported a maximal outward I_K1_ density in guinea-pig ventricular myocytes of 3.1 ± 0.1 pA/pF from perforated patch whole cell recordings; our maximal outward Ba^2+^ sensitive I_K1_ elicited during voltage ramps was 4.9 ± 0.4 pA/pF (albeit under different conditions as we did not use perforated patch recording). Taken together these observations are suggestive that the Ba^2+^ ion concentration used in this study can be expected to have very largely inhibited I_K1_, though we do not exclude the possibility that a small fraction of the total available current may have remained unblocked. Any residual unblocked component would have been constant during a given dynamic clamp experiment, as 50 µM Ba^2+^ was present throughout these measurements: the control (WT) condition was established by application of sufficient synthetic I_K1_ to restore APD_90_ and then the only subsequent experimental changes made were to substitute the mutant for WT I_K1_ formulations.

Miake *et al* have previously studied the role of I_K1_ in guinea-pig ventricular AP repolarization and in setting resting potential, through overexpression of Kir2.1 and suppression using a dominant negative Kir2.1 construct, the latter reducing outward I_K1_ by 82.1% (Miake et al., 2003). The mean resting potential of transduced ventricular myocytes was hyperpolarized by 4.4 mV with Kir2.1 overexpression, whilst it was depolarized by 6.7 mV with expression of the dominant negative construct to suppress I_K1_ (Miake et al., 2003). Despite these experimental conditions (transduced, cultured myocytes) differing from those used here, the two studies are in close agreement in the extent of resting membrane potential depolarization with I_K1_ suppression (∼7 and ∼6 mV respectively). Miake *et al* also reported that dominant negative I_K1_ suppression had a greater effect on APD_90_ than on APD_50_, slowing repolarization velocity over this time period (Miake et al., 2003). This is in good agreement with the increased AP triangulation seen in the present study with Ba^2+^ application. Beat-to-beat variability in APD_90_ was not evaluated in that study (Miake et al., 2003), so comparisons with our data are not possible in this regard. However, increased short term variability in rat ventricular AP duration has been reported with I_K1_ inhibition (Skarsfeldt et al., 2016), which is consistent with our results. Thus, the effects of I_K1_ inhibition with 50 µM Ba^2+^ in this study are consistent with anticipated effects of substantial I_K1_ reduction.

Application of synthetic I_K1_ in the presence of Ba^2+^ was able to restore APD_90_ and produce APs of a normal morphology, which then provided a baseline for the investigation of effects of the D172N Kir2.1 mutation. Interestingly, the peak amplitude of outward I_K1_ during terminal repolarization supplied under dynamic clamp in the present study is similar to that observed previously for [K^+^]_o_ sensitive I_K1_ from guinea-pig myocytes under AP clamp (Figure 9c of Li et al., 2000). The profile of WT I_K1_ during late AP repolarization in our experiments was as expected and the profile and timing of this current (evident in the instantaneous I-V plot for WT I_K1_) explain why reduction of I_K1_ predominantly affects late repolarization. The initial rapid outward current component seen during AP depolarization merits some comment, however. As noted in the Results section, a transient outward component of I_K1_ has been reported in guinea-pig and canine myocytes previously (Li et al., 1998; Li et al., 2000). Under AP clamp of ventricular myocytes from both species, the upstroke of the applied AP command elicited a rapid transient I_K1_ [measured as either K^+^_o_-sensitive or Ba^2+^-sensitive current; (Li et al., 1998; Li et al., 2000; Li & Dong, 2010)]. Further experiments on recombinant Kir2.1 channels showed that they are also able to generate an early transient outward current component (Zhang et al., 2009). Such transients are consistent with rapid voltage excursions during the AP upstroke through the membrane potential range within which outward I_K1_ occurs. From this and considering the comparatively fast loop time of our dynamic clamp system (see Methods), we infer that at least a part of the initial outward current at the start of the AP was not artifactual.

### Effects of the D172N Mutation With Dynamic Clamp–Results in Context

The inward rectification of Kir2.1 depends on three positively charged amino acid residues in the second transmembrane domain (D172), and C terminus (E244 and E299) of the channel (Abrams et al., 1996; Xie et al., 2002; Xie et al., 2003). The D172N mutation reduces the voltage dependent block of the channel responsible for inward rectification, thereby increasing outward Kir2.1 current (Abrams et al., 1996; Priori et al., 2005) (and hence I_K1_). In the initial report of SQT3 arising from the D172N mutation, simulations using the Priebe-Beuckelmann human ventricular AP model (Priebe and Beuckelmann, 1998) predicted augmented outward I_K1_ leading to APD abbreviation due to effects arising exclusively during late phase repolarization, leading to accelerated terminal repolarization (Priori et al., 2005). Our own subsequent simulation work (Adeniran et al., 2012) using modifications to the ten Tusscher and Panfilov (2006) human ventricular AP model based on experimental data from WT and D172N channels (El Harchi et al., 2009) showed APD shortening arising late in repolarization with steepening of terminal repolarization. Resting potential values were little altered, however, with less than 1 mV of difference in resting potential between the WT and homozygous D172N condition in our simulations (Adeniran et al., 2012). An independent study, using the O’Hara et al (2011) human ventricular AP model showed very similar effects of the D172N mutation in respect of steepening of late AP repolarization and little effect on resting potential (Deo et al., 2013). Steepening of this final AP repolarization phase was shown in multicellular models to account for the asymmetric, tall T waves seen in the SQT3 proband (Priori et al., 2005; Adeniran et al., 2012). Our dynamic clamp data from guinea-pig ventricular myocytes and hiPSC-CMs are therefore consistent with and supportive of the results from prior AP simulations (Priori et al., 2005; Adeniran et al., 2012; Adeniran et al., 2013; Deo et al., 2013), in showing: APD_90_ abbreviation; abbreviation of the APD_90_-APD_30_ interval; increased maximal repolarization velocity and little change in resting membrane potential. Additionally, our experiments indicated that these changes occurred without any exacerbation of beat-to-beat variability in APD_90_.

Deo and others predicted the effects of the D172N Kir2.1 mutation on ventricular repolarization to be rather less than those of the SQT3 E299V Kir2.1 mutation reported for the first time in their study (Deo et al., 2013). This is entirely consistent with the much more extensive abolition of current rectification produced by the E299V mutation, which leads to augmented I_K1_ over a greater membrane potential range than does D172N (Deo et al., 2013). In turn, this led to dramatic AP shortening when applied under dynamic clamp to hiPSC-CMs (Meijer van Putten et al., 2015). The D172N mutation produces a less extensive AP abbreviation (as shown in this study and Priori et al., 2005; Adeniran et al., 2012; Adeniran et al., 2013; Deo et al., 2013).

The D172N expressing proband and her father were both heterozygous for this Kir2.1 mutation (Priori et al., 2005) and so any arrhythmic substrate *in vivo* is due to heterozygous and not homozygous expression of the mutation. Accordingly, the findings of this study most relevant to the clinical scenario are those with the heterozygotic WT-D172N I_K1_ formulation. In this regard, it is notable that the biggest change in guinea-pig ventricular AP profile occurred when the WT I_K1_ formulation was switched to the heterozygous WT-D172N formulation, with relatively little additional effect of the homozygous D172N formulation. In our experiments on hiPSC-CMs it was also the case that introduction of the heterozygous WT-D172N formulation had a marked effect on repolarization parameters, with relatively modest increases in most effects on further application of the homozygous D172N formulation. Collectively, our results on both cell preparations are in fair agreement with ventricular AP simulations from studies in which D172N I_K1_ produced relatively modest further AP abbreviation in comparison to WT-D172N I_K1_ (Priori et al., 2005; Adeniran et al., 2012; Deo et al., 2013). We found that guinea-pig ventricular APD_90_ was abbreviated over a range of stimulation rates by the D172N-containing I_K1_ formulations, shown by the downward shift of the APD_90_-cycle length relationship in Figure 6. This is also in good agreement with prior simulations, which showed that simulated APD-rate or restitution relationships (plotted as APD against diastolic interval/basic cycle length) for D172N conditions were down-ward shifted compared to that for WT I_K1_ (Priori et al., 2005; Adeniran et al., 2012; Deo et al., 2013). In particular the simulations of Deo et al., showed parallel-downward shifted APD-BCL relationships, with a marked effect of heterozygotic incorporation of the D172N mutation and a relatively modest additional effect of the homozygous condition. The shorter APD values over a range of diastolic intervals have been associated in simulations with abbreviated effective refractory periods (ERPs), which contribute to the proarrhythmic substrate in tissue simulations with the D172N mutation (Adeniran et al., 2012). Simulations incorporating very short diastolic intervals/cycle lengths (Adeniran et al., 2012) showed an additional tendency for increase of steepness of maximal slope of APD-restitution. Whilst our experimentally derived APD-rate relationships encompass a range of rates relevant to human repolarization, the requirement to apply successfully multiple stimulation rates with three different I_K1_ formulations prevented us from including stimulation rates >3 Hz.

The modest decrease (<20%) in guinea-pig ventricular AP maximal upstroke velocity seen with the WT-D172N and D172N I_K1_ formulations appears to correlate with the greater opposing early outward transient component of I_K1_ elicited during the AP upstroke. Although this observation has not been highlighted in prior simulations of the effects of D172N on ventricular electrophysiology (Priori et al., 2005; Adeniran et al., 2012; Deo et al., 2013), prominent outward I_K1_ transients during the ventricular AP upstroke have been observed in some simulations of the effect of the D172N Kir2.1 mutation (Adeniran et al., 2013) and a decrease in ventricular conduction velocity (CV) at rates <107 beats min^−1^ was predicted with the mutation by Adeniran et al. (2012) due to reduced tissue excitability. In simulations at high rates tissue CV actually increased, which was thought to be attributable to ERP abbreviation (Adeniran et al., 2012). Notably, we did not see a similar effect in our experiments on hiPSC-CMs: there was a statistically insignificant increase in maximal upstroke velocity under WT-D172N (i.e., clinically relevant) conditions, an effect that became significant for homozygous conditions. hiPSC-CMs also exhibited a modest hyperpolarization in resting potential when WT I_K1_ was exchanged for heterozygotic or homozygotic D172N I_K1_. It seems likely that these two changes are related and that any effect of hyperpolarizing resting membrane potential on I_Na_ availability may have predominated over any increased early I_K1_ transients, in influencing the AP upstroke velocity. The hyperpolarization of resting potential with D172N-modified I_K1_ in hiPSC-CMs differs both from our results from guinea-pig ventricular myocytes and prior ventricular simulations of effects of the mutation (Priori et al., 2005; Adeniran et al., 2012; Deo et al., 2013). However, simulations of *atrial* effects of the D172N Kir2.1 mutation have shown a modest (up to ∼5 mV) hyperpolarization of resting membrane potential together with an (up to ∼20 V/s) increase in AP maximal upstroke velocity (Whittaker et al., 2017). Although the hiPSC-CMs that we used exhibited ventricular-like APs when supplied with synthetic I_K1_, they were derived from a mixed (as opposed to ventricular-like cell enriched) cell preparation. Further, spontaneously active myocytes typically have highly labile membrane potentials over the diastolic potential range (Irisawa et al., 1993; Hancox et al., 2003) and so it is perhaps not surprising that hiPSC-CM resting potential was more sensitive to mutant I_K1_ formulations than that from adult ventricular myocytes. It is also of interest that in dynamic clamp experiments using hiPSC-CMs to study effects of the E299V Kir2.1 mutation Meijer van Putten et al (2015) observed hyperpolarization of MDP when that gain-of-function mutant I_K1_ formulation was introduced.

### Potential Limitations and Conclusions

Channels underpinning native I_K1_ involve other Kir2.x isoforms in addition to Kir2.1 (Dhamoon & Jalife, 2005) and modifications to I_K1_ in this study were based solely on changes to Kir2.1 function. However, in the human ventricle Kir2.1 transcript dominates (>90%) over other isoforms (Wang et al., 1998) and the profound effect of Kir2.1 suppression on guinea-pig ventricular I_K1_ (Miake et al., 2003) suggests that Kir2.1 is predominantly responsible for I_K1_ in this species as well. It is therefore reasonable to modify I_K1_ according to alterations to Kir2.1 current properties, as has been implemented in prior SQT3 simulation (Priori et al., 2005; Adeniran et al., 2012; Deo et al., 2013; Whittaker et al., 2017) and dynamic clamp (Meijer van Putten et al., 2015) investigations. The scaling of WT-D172N and D172N formulations relative to WT amplitudes based on I-V data in Figure 1A of Adeniran et al (2012) rather than using differences in simulated AP clamp data (Figure 1B of that study) was conservative; larger mutation effects on AP parameters, particularly in the homozygotic condition, might have been observed in dynamic clamp experiments in this study had those larger scaling ratios been used. However, D172N mutation carriers were heterozygotic for the mutation (Priori et al., 2005) and so the homozygotic experimental condition is without direct clinical relevance. Native I_K1_ is sensitive to intracellular [Ca^2+^]_i_ (Zaza et al., 1998) and there would be no dynamic modulation of I_K1_ by [Ca^2+^]_i_ transients for synthetic I_K1_. However, this limitation would apply also to prior simulation studies and it is notable that: (a) the experimental results observed here are concordant with major AP changes predicted from computational modelling (Priori et al., 2005; Adeniran et al., 2012; Adeniran et al., 2013; Deo et al., 2013), and (b) that tissue models incorporating these AP changes recapitulate ECG changes seen in patients and have shown how substrate(s) for re-entrant arrhythmia may arise (Priori et al., 2005; Adeniran et al., 2012). This highlights that, optimally, dynamic clamp experiments can be used together with cell and tissue computational modelling, as the combination of these approaches is likely to provide robust insight into cellular effects of channel mutations, with tissue modelling then allowing evaluation of the tissue arrhythmia substrate(s) that is not possible from *in vitro* single cell experiments alone. In the case of D172N Kir2.1, this provides confidence that the simulated AP changes predicating prior predictions of increased temporal vulnerability and reduced minimal substrate size for sustaining reentry in this form of SQT3 are likely to be valid (Adeniran et al., 2012). Another potential limitation, which is inherent in the dynamic clamp approach, is that synthetic currents are not amenable to direct pharmacological modulation, so that it is not possible to investigate directly effects of acute selective pharmacological inhibition on gain-of-function mutations. Nevertheless, our study provides further evidence of the value of dynamic clamp in interrogating functional consequences of Kir2.1-related channelopathies (Meijer van Putten et al., 2015), whilst highlighting the possibility that results from mature adult cardiomyocytes and hiPSC-CMs are likely to be similar, but may not be identical.

## Data Availability

The original contributions presented in the study are included in the article/supplementary material, further inquiries can be directed to the corresponding author.
